# Males of the parasitoid wasp, *Nasonia vitripennis*, can identify which fly hosts contain females

**DOI:** 10.1098/rsos.211865

**Published:** 2022-01-26

**Authors:** Garima Prazapati, Ankit Yadav, Anoop Ambili, Abhilasha Sharma, Rhitoban Raychoudhury

**Affiliations:** ^1^ Department of Biological Sciences, Indian Institute of Science Education and Research (IISER) Mohali, Knowledge City, Sector- 81, Manauli P.O. 140306, India; ^2^ Department of Earth and Environmental Sciences, Indian Institute of Science Education and Research (IISER) Mohali, Knowledge City, Sector- 81, Manauli P.O. 140306, India

**Keywords:** *Nasonia*, chemical, mating, fitness, wasps, parasitoid

## Abstract

The reproductive success of a male is limited by the number of females it can mate with. Thus, males deploy elaborate strategies to maximize access to females. In *Nasonia*, which are parasitoids of cyclorrhaphous flies, such reproductive strategies are thought to be restricted to competition among males for access to females in the natal patch. This study investigates whether additional strategies are present, especially the capability to identify which fly hosts contain adult females inside. Behavioural assays revealed that only one out of the four species, *N. vitripennis,* can distinguish which hosts specifically have adult female wasps, indicating a species-specific reproductive strategy. Results of gas chromatography-mass spectrometry analyses and behavioural data suggest that female-signature cuticular hydrocarbons (CHCs) are used as chemical cues, possibly emanating from within the host puparium. Further assays indicated that *N. vitripennis* males can also detect differences in the intensities of female-signature CHCs, giving them the capability to seek out hosts with maximum number of females. This study uncovers a previously unknown reproductive strategy in one of the most widely studied parasitoid wasps.

## 1. Introduction

In most sexually reproducing organisms, male reproductive success is limited by the number of fertile females it can mate with [1]. By contrast, female reproductive success is mainly limited by the number of eggs available [1]. This difference necessitates distinct reproductive strategies for both [2]. The ideal reproductive strategy for a male involves rapid sexual maturation and access to many fertile females [3]. Thus, males have evolved several reproductive strategies. One such strategy seen in some parasitoid wasps is protandry, where males emerge before the females to gain better access to them [4–7]. However, male parasitoid wasps experience intense competition to access emerging females as mating is usually restricted to the natal host patch [8,9]. Therefore, males are expected to develop additional reproductive strategies to maximize individual fitness, many of which involve using chemical cues [10]. Males use trail sex pheromones of females in *Aphelinus asychis* [11], *Aphytis melinus* [12] and *Trichogramma brassicae* [13] or attract them by pheromone markings like in *Urolepis rufipes* [14]. Since parasitoid wasps usually mate in the natal host patch [9], short-range chemicals like cuticular hydrocarbons (CHCs) can also act as sex pheromones [15,16]. Further, the use of the complete CHCs profiles, as well as single compounds, has been shown to elicit behavioural responses. These include branched hydrocarbons involved in mate recognition in *Ooencyrtus kuvanae* [17], in host recognition in *Holepyris sylvanidis* [18] and in *Dibrachyscavus* [19] as well as in Lariophagus *distinguendus* [20] as contact sex pheromones. Branched hydrocarbons also work synergistically with the straight chain hydrocarbons as contact sex pheromones in *Urolepsis rufipes* [21]. Obtaining mates may also involve chemical cues emanating from the hosts themselves. For instance, the parasitoid wasp *Cephalonomia tarsalis* uses host-associated sex pheromones [22], whereas the wasp *Lariophagus distinguendus* [23] uses other host-derived cues. Similarly, some braconid wasps use host-induced plant volatiles [24] as well as sex pheromones [25] to locate mates. Moreover, the ability of parasitoid wasps to use vibratory cues has been demonstrated in the wasp *Pimpla disparis* [26] where such cues attract males to the gypsy moth hosts.

This study tries to identify additional reproductive strategies of the males of the genus *Nasonia* (Hymenoptera: Chalcidoidea: Pteromalidae) which comprises four species, *N. vitripennis*, *N. longicornis*, *N. giraulti* and *N. oneida* [27]. Like all Hymenoptera, *Nasonia* is haplodiploid, with diploid females developing from fertilized eggs, and haploid males developing from unfertilized eggs through arrhenotoky. The female wasp parasitizes cyclorrhaphous fly pupae by drilling through the host's puparium with its ovipositor to paralyse the fly pupa by injecting it with venom [28]. Females then proceed to lay 15–30 eggs on the surface of the fly pupa inside the puparium [29]. This holometabolous life cycle, from eggs to adults, happens inside the host, and the adults emerge by chewing a hole through the puparium. The hosts that *Nasonia* females parasitize are available as a patchy resource and each female often parasitizes multiple hosts [30]. Despite a female-biased offspring sex ratio [29], the males usually emerge first from their natal host and mark surface territories with sex pheromones which can attract emerging females [31].

However, several biological features of *Nasonia* indicate that males may be under strong selection pressure to evolve additional strategies to seek out females beyond their natal host. As males are brachypterous by birth [29], they are confined to find mates within their immediate vicinity. Females, which usually mate only once [32], quickly fly away in search of new hosts [33].

Males have been known to aggressively defend the emergence holes in the puparium, due to intense competition for emerging females from other males [34]. This intra-sexual aggression may also trigger additional strategies, like the ability to seek other hosts from which new adult females may emerge. Such a phenomenon has been postulated to significantly increase male fitness [35], especially if the males perceive cues emanating from hosts that yield adult females [36]. Shuker *et al.* [37] found that *N. vitripennis* males were significantly more likely to stay on a patch which contains hosts which are about to yield adult females. Such behaviour would increase the likelihood of asymmetrical local mate competition. However, it is also known that *N. vitripennis* males with prior experience of successful mating spend significantly more time on hosts with adult females inside [38]. This can reduce a male's drive to search the patch for other hosts with adult females inside, thereby significantly altering the behaviour of males toward any future interactions in the patch [38]. Therefore, it is not clear whether a virgin *N. vitripennis* male has the ability to ‘recognize’ hosts containing adult females inside. Moreover, what also remains unknown is the presence of this ability in other *Nasonia* species and the nature of cues used by males to detect such hosts.

This study is a comprehensive investigation of a potential reproductive strategy in the four *Nasonia* species. By providing individual males with a choice of different fly hosts, we tested whether males can distinguish the ones that may contain females from others that do not. In a series of subsequent experiments, we also determined the nature of the cues (vibratory, visual or chemical) involved and found a species-specific reproductive strategy that depends on the males' ability to detect female-specific signature CHCs emanating from the hosts.

## 2. Material and methods

### 2.1. *Nasonia* strains used

The strains of the four *Nasonia* species used were NV-IPU08 (*N. vitripennis* from Punjab, India), NL-MN8501 (*N. longicornis*), NG-RV2XU (*N. giraulti*) and NO-NY11/36 (*N. oneida*). They were reared in a 24 h light cycle, at 25°C, with 60% relative humidity and had an average life cycle of 14 days for *N. vitripennis*, 14.5 days for *N. longicornis*, 15 days for *N. giraulti* and 16 days for *N. oneida*. The different life-stages include egg (1–2 days), larva (2–7 days), pupa (8–13 days) and adult (14–16 days). Single adult females were allowed to parasitize two fly hosts for 48 h. The obtained parasitized hosts were either kept for wasp emergence or used in the experiments as required.

### 2.2. Fly hosts used

All *Nasonia* cultures were raised on pupae of the fly, *Sarcophaga dux,* which has a life cycle of 11 days at 25°C. The larvae were fed with chicken liver, and the fly pupae were stored at 4°C. These fly pupae, kept at 4°C for less than or equal to 48 h, were designated as ‘unparasitized’ hosts. The parasitized hosts that contained larval wasps were abbreviated as HwL (hosts with larval wasps), and those with eclosed (but un-emerged) adult wasps were designated either as HwAMF (hosts with both adult males and females) or as HwAM (hosts with adult males). Both HwL and HwAMF were obtained from parasitization by mated females, and HwAM was obtained from parasitization by virgin females. Before being used in the experiments, the absence of any emergence holes made by the adult wasps was noted. After each assay, all the parasitized hosts were cracked open to confirm if they contained the requisite sex, developmental stage, alive or dead wasps.

### 2.3. Behavioural assay

An equal-choice cafeteria arena was set up to test for male preference. This arena was fashioned out of a glass Petri plate and a white sheet of paper with two concentric circles (outer 9 cm and inner 5 cm diameter) drawn out with the entire area equally divided into six sections (electronic supplementary material, figure S2). Each Petri plate was cleaned with ethanol, HPLC grade n-hexane and autoclaved before being used. Autoclaved distilled water was also added along with the circumference of the Petri plates to prevent the males from escaping the arena. This set-up was placed on a wooden platform with a 5-watt LED lamp placed 30 cm above it. Males were dropped in the centre of the arena (inner circle) and their behaviour was video-recorded (Logitech C615 HD webcam) for 4 min at 25°C ± 1°C. Each assay, i.e. every data point, was obtained by using a fresh Petri plate with a fresh set of hosts (three hosts of two types, placed alternately). A naive male was randomly selected from all-male broods and used in all the experiments to avoid any possible sensory bias from co-development with females.

All parasitized hosts were handled with separate forceps that were washed with 70% ethanol, HPLC grade *n*-hexane and then autoclaved. Male preference for either type of host (electronic supplementary material, figure S2) was quantified by the average time spent on each host. The time was counted from the moment a male climbed on a host and continued until dismounting and abandoning it. Each male was used only once. The sample sizes obtained for each type of assay were from *N* = 15–26. All the video data obtained for the behavioural assays have been uploaded on https://www.youtube.com/channel/UCBh3wyHrAty7dvcLNX6HeOw/videos. Statistical analyses for the behavioural assays were done in RStudio, v. 1.2.5033 [39]. Shapiro–Wilk test [40] was used to test for normality using the *stats* package of R v. 4.1.0 [41]. The obtained data tested negative for normality, hence, the Wilcoxon signed-rank test (significant at *p* < 0.05) was used to assess male preferences. Effect sizes (*r*) were calculated from the Z-statistic obtained from the Wilcoxon signed-rank test using the *stats* package. Wilcoxon effect size (*r*) values range from *r* = 0.1 to less than 0.3 (small effect), *r* = 0.3 to less than 0.5 (moderate effect) and *r* ≥ 0.5 (large effect). Boxplots were made by using the *ggplot2* package [42].

### 2.4. Determination of the use of vibratory cues

To remove any possible vibratory cues emanating from live wasps, HwAMF were kept at −80°C for 2 h to kill the adult wasps inside. Hosts were used for the assay within 2 h of being brought back to room temperature, which was confirmed by using an LCD digital I.R. temperature laser gun (Dual Laser Optical Focus Temperature Gun, NUB8580). *N. vitripennis* males were given a choice between such freeze-killed HwAMF hosts (with dead adult wasps) and those with live adult wasps inside.

### 2.5. Determination of the use of visual cues

To test whether males can visually discriminate between the given host type, they were given a choice in the puparial halves of hosts exhibiting differential darkening. Such puparial halves were obtained from the anterior end (electronic supplementary material, figure S4) of unparasitized hosts at different developmental ages–one with fly pupa and the other with the adult fly. Males were also tested for their visual discriminating ability towards puparial halves of parasitized (HwL) and unparasitized (control) hosts with a similar degree of darkening. Puparial halves of HwL were obtained by embedding the posterior end of an unparasitized host within a foam plug in a vial to ensure that chemical cues left by the female, if any, remain localized on the anterior end. Single adult females were introduced in half of these vials for parasitization while the rest served as control. The parasitized hosts were taken out of the plugs after 48 h and checked for the presence of eggs (localized at the head of the fly pupa) by carefully cracking open the anterior end to collect the puparial halves. Males were given a choice between puparial halves from such parasitized hosts (HwL) and those from the control.

### 2.6. Fractionation of chemical cues by column chromatography

Fifty intact parasitized hosts (HwAMF) were dipped in 3 ml of HPLC grade *n*-hexane (Merck Corp.) in a glass vial at room temperature for 5 min. This relatively short time was used to minimize chemicals leaching from within the hosts. The extract was pipetted out in a separate glass vial and concentrated to 100 µl under a gentle nitrogen stream. To obtain the desired fractions, the extract was poured onto a 3 cm column, fashioned out of a glass Pasteur pipette (inner diameter = 0.7 cm) packed with baked glass wool (Sigma Aldrich) and activated silica gel (100–200 Mesh; Merck Millipore, baked at 250°C for 12 h). The column was pre-conditioned by washing it with 1 ml of *n*-hexane. The elution of non-polar fraction was done with 1 ml of *n*-hexane followed by the elution of the polar fraction with a 1 ml solution of dichloromethane and methanol (9 : 1). Both the polar and non-polar fractions were concentrated under a gentle nitrogen stream and reconstituted in a 50 µl (1 host equivalent per µl) mixture of dichloromethane and methanol (9 : 1) and *n*-hexane, respectively, in separate glass vials.

For the behavioural assay, three puparial halves (obtained from anterior ends of unparasitized hosts) were separately poured with 2 µl (2 host equivalent extract) of the non-polar fraction, while the rest were poured with 2 µl of *n*-hexane. All puparial halves were air-dried for a minute before introducing the male into the arena for the assay. Similarly, male preference was also tested towards the polar extract. The same column chromatography method was used to isolate the non-polar fractions from fifty intact HwAM and unparasitized hosts.

The CHCs fractions from adult males and females (separated by sex in their pupal stage of development) were obtained by dipping 50 individuals of each in 500 µl of HPLC grade *n*-hexane (Merck Corp.) for 10 min, in separate glass vials. These extracts were transferred to a fresh set of glass vials, but only the non-polar fraction (enriched for CHCs) was eluted with 1 ml of *n*-hexane through the column chromatography method described above. These extracts were then carefully concentrated under a gentle nitrogen stream and reconstituted in 250 µl (1/5 wasp equivalent per µl) of *n*-hexane for both male and female extracts, separately. Male preference for both extracts was tested in separate experiments, where three unparasitized hosts were separately poured with 5 µl (1 wasp equivalent extract) of the extract, while the remaining were poured with 5 µl of *n*-hexane to serve as a control. Another set of extractions of adult female CHCs was done using the same protocol and reconstituted to a final concentration of 50 µl (1 wasp equivalent per µl), for use as 5× concentrated fraction of CHCs. 5 µl (5 wasp equivalent extract) of this 5× concentrated fraction (50 µl) was poured on three unparasitized hosts and 5 µl of 1× concentrated fraction (250 µl) of female CHCs was poured on the rest.

### 2.7. Identification of the chemical compounds by gas chromatography-mass spectrometry

Non-polar fractions from HwAMF, HwAM and unparasitized hosts were enriched through column chromatography described above. Two microliters (1 host equivalent per µl) of the obtained fractions from each were separately injected (split-less mode) into a gas chromatograph coupled with a mass spectrometer (Agilent 7890B, 5977C GC-MS) equipped with a capillary column, HP-5MS (Agilent J&W) of 30 m length, 0.25 mm internal diameter and 0.25 µm thickness, and were operated at the electron impact ionization mode of 70 eV. The quadrupole temperature was kept at 150°C, while the inlet and the auxiliary line temperature were maintained at 320°C. Helium was used as the carrier gas with an average linear velocity of 36.2 cm s^−1^. The oven temperature was started at 40°C, with a hold of 5 min, and increased to 300°C at the rate of 4°C/min followed by a final hold of 25 min. A total of five replicates were analysed for each of the samples (unparasitized hosts, HwAM and HwAMF). Similarly, 2 µl (1 wasp equivalent per µl) extract from adult males and females (four replicates of each) were also analysed through gas chromatography-mass spectrometry (GC-MS) under the same conditions. The compounds were identified according to their characteristic diagnostic ions and resulting mass spectra [43–45]. Methyl-branched hydrocarbons were identified by their characteristic diagnostic ions resulting from fragmentations at the branching point, the extracted ion chromatogram (EIC-*m/z*) and the linear retention index values from previously published data [44–46]. An *n*-alkane (C8-C40, SUPELCO) standard was also analysed under the same conditions to calculate the relative retention indices of the identified CHCs. Peaks were identified and integrated with Mass Hunter Workstation Software vB.08.00 (Agilent Technologies). The composition of the obtained chemical profile was analysed by obtaining the relative abundances of the individual compounds by relating the peak area for each compound to the total peak area for each run. The magnitude of difference in the relative abundances of each compound, between samples, was measured by Cohen's *d* [47] where a value less than 0.2 indicates a negligible effect size and *d* > 0.5 indicates a large effect size [47]*.* An analysis of similarity percentages (SIMPER), using Bray–Curtis dissimilarities as a distance measure, was done to know the contribution of all the compounds contributing towards the dissimilarity observed between the samples. For redundancy analysis (RDA), the relative abundances of all compounds (electronic supplementary material, table S1) were log-transformed (centred log-ratio transformation) according to the following formula [48]:

$$\mathrm{clr}(x)=\left(\log\left(\frac{x_{1}}{g(x)}\right),\ldots ,\;\log\;\left(\frac{xD}{g(x)}\right)\right),$$

where *x* represents the composition vector, *g*(*x*) the geometric mean of the composition *x* and *xD* the Euclidean distances between the individual variables. As the data included some undetectable compounds in some samples, a small constant was added to all values before transformation [49]. The centred log-ratio transformation, RDA and the SIMPER analysis were carried out in the software PAST v. 4.06b [50].

## 3. Results

### 3.1. *Nasonia* males can detect parasitized hosts

**Figure 1.**
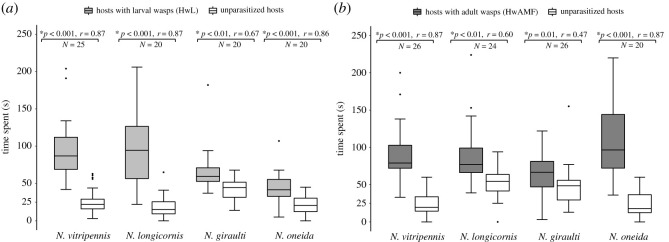
*Nasonia* males can detect parasitized hosts. (*a*) Average time spent by males of all the four species on parasitized hosts containing larval wasps (HwL) versus unparasitized ones. Males of all four species spent significantly more time on parasitized hosts indicating their ability to detect hosts with larval wasps inside. (*b*) Average time spent by males on parasitized hosts containing adult wasps of both sexes (HwAMF) and unparasitized ones. Males of all the four species again spent significantly more time on parasitized hosts with adult wasps inside. In the boxplots, the horizontal bold line represents the median, boxes represent 25% and 75% quartiles, whiskers denote 1.5 interquartile ranges and black dots depict outliers and asterisk denotes statistical significance at *p* < 0.05 according to Wilcoxon signed-rank test. Wilcoxon effect size (*r*) values range from *r* = 0.1 to less than 0.3 (small effect), *r* = 0.3 to less than 0.5 (moderate effect) and *r* ≥ 0.5 (large effect).

When given a choice between three parasitized hosts containing wasp larvae (HwL) and three unparasitized ones, males of all the four *Nasonia* species can detect the parasitized ones as they spend significantly more time on them (figure 1*a*; *p* < 0.001, *r* = 0.87 for *N. vitripennis*; *p* < 0.001, *r* = 0.87 for *N. longicornis*; *p* < 0.01, *r* = 0.67 for *N. giraulti* and *p* < 0.001, *r* = 0.86 for *N. oneida*). However, each HwL has larval wasps inside it which are several days away from adult wasp eclosion and can extend well beyond the life span of the males. Hence, to test whether males can identify parasitized hosts which contain eclosed adults (HwAMF), a choice was given between such hosts and the unparasitized ones. As figure 1*b* shows, males of all the four species spend significantly more time on HwAMF (*p* < 0.001, *r* = 0.87 for *N. vitripennis*; *p* < 0.01, *r* = 0.60 for *N. longicornis*; *p* = 0.01, *r* = 0.47 for *N. giraulti* and *p* < 0.001, *r* = 0.87 for *N. oneida*). Thus, *Nasonia* males can detect parasitized hosts which contain either larval or adult wasps. This ability is not influenced by any inherent directional bias as control experiments with six unparasitized hosts across the four species showed no evidence of bias (electronic supplementary material, figure S3).

### 3.2. *Nasonia vitripennis* males use chemical cues to detect parasitized hosts

**Figure 2.**
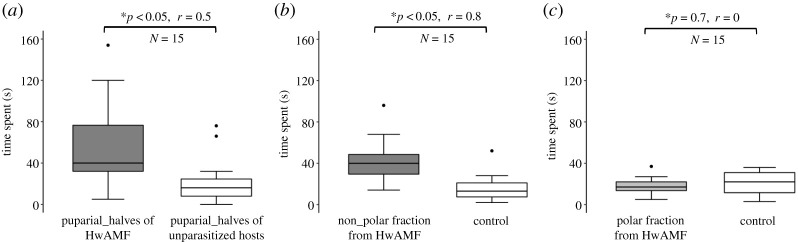
*Nasonia vitripennis* males use chemical cues to detect parasitized hosts. (*a*) Males prefer puparial halves of HwAMF (hosts containing adult wasps) over those of unparasitized fly hosts (containing adult fly) as they spent more time on puparial halves of HwAMF. (*b*) Males use chemical cues as they spent more time on the non-polar fraction of the extract (enriched for CHCs) from HwAMF versus control (poured with pure solvent). However, males show no preference (*c*) towards the polar fraction of the extract from HwAMF. Thus, the source of chemical cues used by the *N. vitripennis* males is the non-polar fraction which is enriched for CHCs. In the boxplots, the horizontal bold line represents the median, boxes represent 25% and 75% quartiles, whiskers denote 1.5 interquartile ranges and black dots depict outliers and asterisk denotes statistical significance at *p* < 0.05 according to Wilcoxon signed-rank test. Wilcoxon effect size (*r*) values range from *r* = 0.1 to less than 0.3 (small effect), *r* = 0.3 to less than 0.5 (moderate effect) and *r* ≥ 0.5 (large effect).

We first tested whether vibratory cues and visual cues (in the form of pupal darkening [51]) are used by the males to detect parasitized hosts with adult males and females inside (HwAMF). As electronic supplementary material, figure S5 shows, neither of these cues is utilized. However, males prefer puparial halves of parasitized hosts (HwL) over the unparasitized ones, even though both exhibit a similar level of darkening (electronic supplementary material, figure S5C). Therefore, we tested whether chemical cues are used to detect parasitized hosts. As the puparium of a host is a porous structure [52], males can in principle perceive any chemical cues left behind by the females during parasitization or can detect those emanating from the host itself. When given a choice between puparial halves of HwAMF and unparasitized hosts (containing adult fly), males spent significantly more time (figure 2*a*; *p* < 0.05, *r* = 0.57) on the puparial halves of HwAMF indicating that either some chemical cues deposited by the parasitizing female persist till the emergence of the adult wasps, or males perceive additional cues emanating from the adult wasps within HwAMF.

To further confirm the use of chemical cues as a major source of discrimination, the polar and non-polar fractions of the chemical extract from intact HwAMF hosts were used to test male preference. As figure 2*b* shows, males prefer the non-polar fraction (*p* < 0.01, *r* = 0.81) over the polar fraction (figure 2*c*; *p* = 0.7, *r* = 0.07), indicating that the non-polar fraction containing the CHCs is the chemical cue used by the males to detect parasitized hosts.

### 3.3. *Nasonia vitripennis* males can distinguish parasitized hosts containing adult female wasps

**Figure 3.**
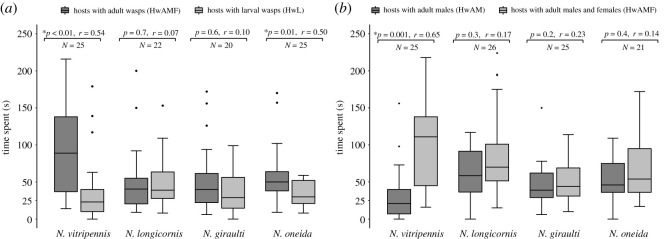
*N. vitripennis* males can distinguish parasitized hosts containing adult female wasps. (*a*) Average time spent by males of all four species on parasitized hosts containing adult wasps of both sexes (HwAMF) and those containing larval wasps (HwL). Males of *N. vitripennis* and *N. oneida* can distinguish between HwAMF over HwL, whereas *N. longicornis* and *N. giraulti* cannot. (*b*) *N. vitripennis* males can distinguish between hosts containing adult wasps of both sexes (HwAMF) over those containing all-male adult broods (HwAM). *N. longicornis, N. giraulti* and *N. oneida* do not show this capability. Thus, only *N. vitripennis* males can distinguish the hosts that will yield adult females. In the boxplots, the horizontal bold line represents the median, boxes represent 25% and 75% quartiles, whiskers denote 1.5 interquartile ranges and black dots depict outliers and asterisk denotes statistical significance at *p* < 0.05 according to Wilcoxon signed-rank test. Wilcoxon effect size (*r*) values range from *r* = 0.1 to less than 0.3 (small effect), *r* = 0.3 to less than 0.5 (moderate effect) and *r* ≥ 0.5 (large effect).

Male were tested for their ability to distinguish between HwAMF and hosts with larval wasps inside (HwL) since detecting HwL (figure 1*a*) adds little to the reproductive success of a male. While males of *N. vitripennis* (figure 3*a*; p < 0.01, *r* = 0.54) and *N. oneida* (*p* = 0.01, *r* = 0.50) show a preference for HwAMF, the males of *N. longicornis* (*p* = 0.7, *r* = 0.07) and *N. giraulti* (*p* = 0.6, *r* = 0.10) do not.

Since virgin females lay only unfertilized eggs (giving rise to all-male broods), the ability to distinguish hosts containing adult wasps adds to the male reproductive success only if a male can distinguish hosts that yield adult females (HwAMF) from those that yield all-male broods (HwAM). Only *N. vitripennis* males show a significant preference (figure 3*b*) for HwAMF (*p* = 0.001, *r* = 0.65), while the other three species do not distinguish between them (figure 3*b*; N*. longicornis*, *p* = 0.3, *r* = 0.17; *N. giraulti*, *p* = 0.2, *r* = 0.23; *N. oneida*, *p* = 0.4, *r* = 0.14). Thus, *N. vitripennis* males can recognize the hosts that yield adult females.

### 3.4. What are the sources of the chemical cues used by *Nasonia vitripennis* males to detect HwAMF?

The source of the chemical cues (CHCs) used by *N. vitripennis* males to identify HwAMF can be any chemical deposited by the parasitizing female, the host itself, or a combination of both. As figure 3*b* shows, males can distinguish HwAMF from HwAM, indicating the presence of unique discriminatory cues in HwAMF. One possibility could be a difference in the progeny sizes between HwAMF and HwAM, where larger broods in the former can result in higher intensity of cues. However, no significant difference in family sizes was found between them (electronic supplementary material, figure S1).

**Figure 4.**
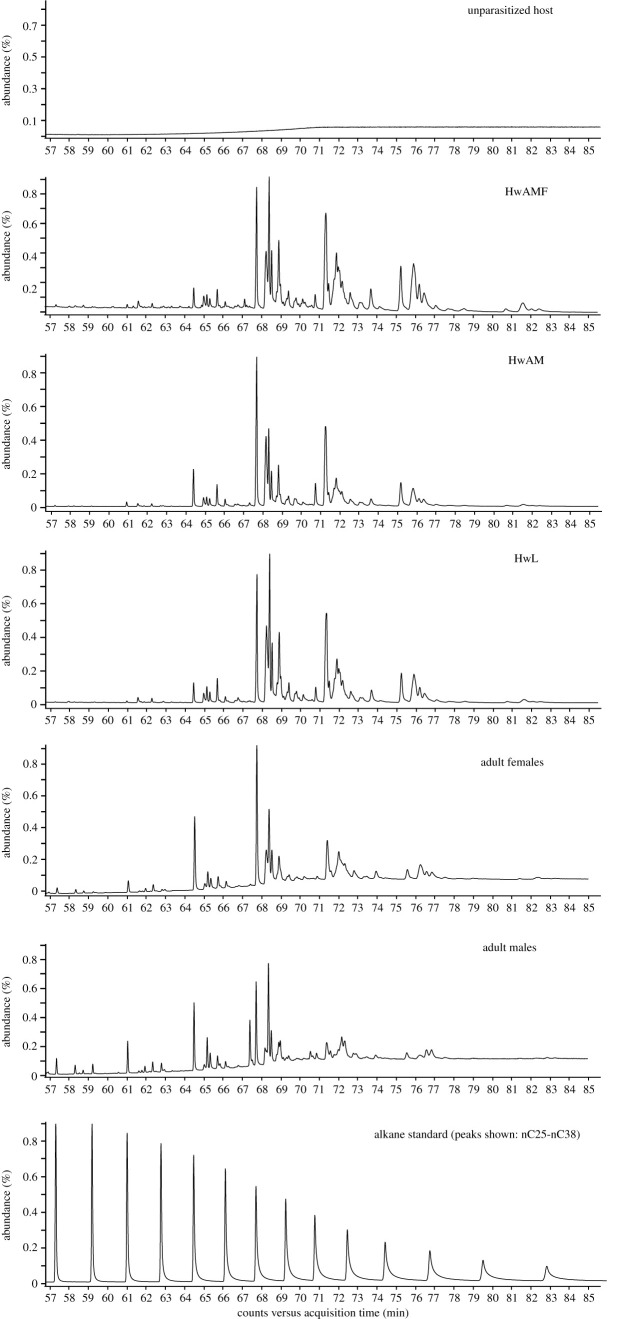
Total-ion chromatogram obtained from different samples. Representative figure of the peaks identified in the chemical profile of the host samples HwAMF, HwAM as well as adult females and males. All the samples share the same 50 compounds, except five compounds unique to the host samples (denoted by Δ in electronic supplementary material, table S1).

The GC-MS analyses of the chemical profiles of both HwAMF and HwAM revealed an array of long-chain saturated and unsaturated hydrocarbons with carbon chain lengths ranging from *n*C25 to *n*C37 (electronic supplementary material, table S1). By contrast, the chemical profile of unparasitized hosts revealed no such compounds (figure 4). This indicates that the primary source of the CHCs is not just the host, but another source in HwAMF. The source of CHCs can be explained by eclosed adults inside HwAMF and HwAM. Since female CHCs are known to attract *N. vitripennis* males [44,45] (electronic supplementary material, figure S6 C; *p* < 0.01, *r* = 0.78), we confirmed the males' preference towards female CHCs by giving them a choice between unparasitized hosts poured with CHCs extract from adult females and those poured with CHCs extract from adult males (electronic supplementary material, figure S6A; *p* < 0.01, *r* = 0.65). However, males spend nearly an equal amount of time on hosts poured with adult male CHCs and those poured with the solvent control (electronic supplementary material, figure S6B; *p* = 0.2, *r* = 0.25), indicating that they perhaps only use the female CHCs profile to distinguish HwAMF from other hosts. Moreover, males prefer hosts poured with a higher intensity of female CHCs (electronic supplementary material, figure S6D; *p* = 0.01, *r* = 0.49). This suggests that the males use both the type (female CHCs) and the intensity of chemical cues for host discrimination.

### 3.5. Which CHCs component do the *Nasonia vitripennis* males use to detect HwAMF?

Any unique compound(s) in the chemical fraction obtained from HwAMF would be an ideal candidate for the discriminating cue; however, no compositional variation exists between the chemical profiles of HwAMF and HwAM (figure 4), as all the 55 identified compounds are present at varying relative abundances in both (electronic supplementary material, table S1). Seventy-six per cent of the profile is made up of methyl-branched alkanes, containing one to four methyl groups, with the rest being *n*-alkanes and mono-alkenes. An RDA shows a clear separation between the HwAMF and HwAM profiles (*R*^2^ adj. = 0.46; electronic supplementary material, figure S7). To identify the differences which reflect the female-specific signature, a comparison was done between the host (HwAMF and HwAM) profiles and the adult sex-specific wasp profiles (electronic supplementary material, table S1). The chemical profiles of the adults share 50 out of the 55 compounds present in HwAMF and HwAM (electronic supplementary material, table S1). However, a SIMPER analysis revealed that the five CHCs unique to the hosts together contribute to only 1.3% of the total dissimilarity (18.47) between them (electronic supplementary material, table S2) and were excluded from further analysis.

Since males prefer HwAMF to HwAM (figure 3*b*) and female CHCs over male CHCs (electronic supplementary material, figure S6A), we searched for the putative female-signature in HwAMF by comparing the relative abundances of individual compounds against HwAM. Out of the 50 compounds common to both, 35 have a significantly higher relative abundance (Cohen's *d* > 0.5) in HwAMF than in HwAM. Out of these 35 compounds, nine compounds (methyl alkanes with carbon chain length > *n*C30) are also significantly higher (Cohen's *d* > 0.5) in adult females than in adult males (table 1). Further, these nine compounds together account for 19.8% of the total dissimilarity (SIMPER analysis) between adult females and males (electronic supplementary material, table S3) and 24.9% of the total dissimilarity between HwAMF and HwAM (electronic supplementary material, table S2), respectively. The compound MeC35 (15-; 13-; 11-) alone contributes to 7% dissimilarity between the host (HwAMF and HwAM) as well as the adult wasp profiles. Therefore, these nine compounds, acting individually or together, can be the candidate female-signature CHCs in HwAMF.

**Table 1.** List of female-signature CHCs: A total of nine compounds make the putative female-signature CHCs from HwAMF. These were found to have a significantly higher (Cohen's *d* > 0.5) relative abundance in HwAMF over HwAM, similar to that found in the adult females over males. Percentage contribution to dissimilarity between the chemical profiles of HwAMF and HwAM is given for all nine compounds, according to the results of the SIMPER analysis. For actual values of relative abundances of the compounds, refer to the complete list of identified CHCs in the electronic supplementary material, table S1.

s. no.	compound name	type	effect size (Cohen's *d*)		contribution to dissimilarity (%)	
			between HwAMF and HwAM	between adult females and males	between HwAMF and HwAM	between adult females and males
1	MeC31 (3-)	monomethylalkanes (with carbon chain length > *n*C30)	5.4	1.0	3.1	0.8
2	MeC33 (5-)		0.9	4.2	0.8	0.9
3	MeC35 (15-; 13-; 11-)		2.3	4.6	7.0	7.1
4	MeC37 (15-; 13-)		1.2	5.5	2.2	1.6
5	DiMeC33 (11,15-; 11,21-)	dimethylalkanes (with carbon chain length > *n*C30)	4.9	5.8	4.3	5.7
6	DiMeC33 (7, 19-; 7, 23-)		7.0	2.5	2.6	0.9
7	DiMeC33 (3,17-; 3,15-)		4.3	0.6	1.1	0.8
8	TetraMeC31 (3,7,11,15-)	tetramethylalkanes (with carbon chain length > *n*C30)	5.2	1	0.8	0.5
9	TetraMeC33 (3,7,11,15-)		6.8	2.4	3.1	1.4

## 4. Discussion

Our results show that the males of all four *Nasonia* species can detect parasitized hosts containing larval as well as adult wasps (figure 1). However, only *N. vitripennis* showed the pronounced effect of this phenotype by recognizing the hosts containing adult female wasps. To achieve this, males presumably use chemical cues in the form of female-signature CHCs emanating from the hosts. *Nasonia* uses several chemical cues during courtship [32,44] and species recognition [44,45,53]. These chemical cues include cuticular lipids acting as contact sex pheromones and other as yet unknown semiochemicals [54]. However, what stands out is the ability of the males to use the female-specific chemical signature from the hosts even before female emergence. Thus, it shows the presence of a previously uncharacterized reproductive strategy in *N. vitripennis* males. A similar reproductive strategy is found in male *Heliconius erato* butterflies which get attracted to the odour of female pupal enclosures and sit on them until eclosion [55]. The males of *H. charitonia* often breach the pupal integument and copulate with the female pupa [56], even before its emergence. Male *Hypoponera opacior* ants can also gain similar reproductive success by sitting on top of the queen cocoons with their genitalia inserted for several hours [57]. *N. vitripennis* represents one of the first examples of such reproductive strategies in parasitoid wasps.

It is also notable that the other three *Nasonia* species do not share the reproductive strategy of *N. vitripennis*. This could possibly be due to within-host mating [58], where mating happens within the fly host before emergence and removes the necessity of additional reproductive strategies. This can perhaps explain why *N. giraulti* does not show this reproductive strategy as it exhibits nearly complete within-host mating. However, *N. longicornis and N. oneida* also do not show this strategy despite exhibiting intermediate to low rates of within-host mating [58]. A crucial biological feature that differentiates *N. vitripennis* from the other three species is their progeny sex ratio. While all *Nasonia* species show pronounced female-biased sex ratios due to local mate competition, *N. vitripennis* shows the least skew while *N. giraulti* shows the highest [59] (electronic supplementary material, figure S1), resulting in the production of comparatively more males in *N. vitripennis*. Whether the presence of more males per host and the absence of within-host mating in *N. vitripennis* is the cause of this strategy or the effect, remains to be investigated. However, what is clear is that the presence of this reproductive strategy is correlated with the high degree of site-fidelity seen in *N. vitripennis* males, much more than *N. giraulti* and *N*. longicornis [34,60], where they do not disperse after emerging from the host. Another consequence of this ability is that it can potentially result in increased male–male conflict, triggering selection for more aggressive male behaviour, as several males can converge on HwAMF in a patch, both for access to females and territoriality. There is some evidence that this could have happened in *N. vitripennis* males as they are the most aggressive among the four species [34,60].

A male *N. vitripennis* can detect variations of the individual CHCs in HwAMF despite compositional uniformity of CHCs with HwAM. These female-specific CHCs can presumably be perceived by the males when they come in contact with the host puparium, as it is a porous structure [52]. We hypothesize a specific list of compounds serving as female-specific signature (table 1) which awaits further empirical validation, preferably with absolute concentrations of the individual compounds. As shown in the electronic supplementary material, figure S6, this reproductive strategy can also extend to a male's capability of distinguishing between different intensities of the female-specific CHCs. This further underscores a male's ability to find hosts, even in a patch, with varying numbers of females inside. Shuker *et al.* [37] showed that mated males are more likely to stay in patches that have hosts with female-biased and intermediate sex ratios over others [37]. Assuming that the cues increase in intensity with the number of females inside a host, a male can now seek out hosts with the maximum number of females, further adding to the reproductive success of a male. Moreover, this ability of males to distinguish between intensities of chemical cues can perhaps explain why males show a preference for parasitized hosts with larvae (HwL) over unparasitized ones (figure 1*a*). Since the analysis of the non-polar fraction from the unparasitized hosts gave undetectable levels of any CHCs (figure 4), it is plausible that the males are detecting the chemical cues left behind by the parasitizing female on HwL. But these cues get swamped when they encounter HwAMF as it usually contains several more adult females inside and, therefore, has a higher intensity of female-specific CHCs (electronic supplementary material, figure S6). Moreover, this can also be the reason why *N. oneida* can distinguish HwAMF from HwL (figure 3a).

*Nasoina vitripennis* has a cosmopolitan distribution, while the other three species are endemic to North America where they are also sympatric with *N. vitripennis* [27]. Reports of micro-sympatry [59] among species (development within the same host) suggest the possibility of reproductive interference between the heterospecific adult males and females [30,59]. The impact of this needs to be verified with sympatric North American *N. vitripennis* strains, as the one used here (NV-IPU8) is from India where there are no reports of any other *Nasonia* species.

Males of all the four *Nasonia* species share the ability to distinguish parasitized hosts from unparasitized ones with other parasitoid wasps, like *Pimpla disparis* [29], *Lariophagus distinguendus* [36], *Spalangia endius* [35] and *Cephalonomia tarsalis* [22]. But none of these parasitoids exhibit the reproductive strategy of *N. vitripennis* male, i.e. the ability to detect females while they are still inside the host. The previous study by Shuker *et al.* [37] showed that males are likely to spend more time in a patch containing HwAMF than in patches with HwAM or HwL [37]. However, the present study provides evidence that males possess the ability to distinguish HwAMF from HwAM or HwL in a patch (figure 3). Moreover, males use the female-signature CHCs as chemical cues to distinguish HwAMF from HwAM. Therefore, the present study is one of the first comprehensive reports of *a N. vitripennis* male's ability to recognize hosts that yield adult females. *Nasonia* is one of the best-characterized insect model systems for understanding the chemical and behavioural basis of sexual communication [54]. Moreover, *Nasonia* belongs to the superfamily Chalcidoidea which has an estimated 500 000 species [61], making it one of the most speciose of any animal group. Many of these species share a similar idiobiont lifestyle with *Nasonia*. Even if a fraction of these species share the ability to detect females still inside their hosts, this can be a major reproductive strategy in the animal kingdom.

## Data accessibility

All behavioural assays are available as videos on https://www.youtube.com/channel/UCBh3wyHrAty7dvcLNX6HeOw/videos. The R script used to generate figures is given within the electronic supplementary material. The data are provided in the electronic supplementary material [62].
